# Adverse events in deep brain stimulation: A retrospective long-term analysis of neurological, psychiatric and other occurrences

**DOI:** 10.1371/journal.pone.0178984

**Published:** 2017-07-05

**Authors:** Carsten Buhmann, Torge Huckhagel, Katja Engel, Alessandro Gulberti, Ute Hidding, Monika Poetter-Nerger, Ines Goerendt, Peter Ludewig, Hanna Braass, Chi-un Choe, Kara Krajewski, Christian Oehlwein, Katrin Mittmann, Andreas K. Engel, Christian Gerloff, Manfred Westphal, Johannes A. Köppen, Christian K. E. Moll, Wolfgang Hamel

**Affiliations:** 1 Klinik für Neurologie, Universitätsklinikum Hamburg-Eppendorf, Hamburg, Germany; 2 Klinik für Neurochirurgie, Universitätsklinikum Hamburg-Eppendorf, Hamburg, Germany; 3 Institut für Neurophysiologie und Pathophysiologie, Universitätsklinikum Hamburg-Eppendorf, Hamburg, Germany; 4 Praxis für Neurologie, Gera, Germany; Oslo Universitetssykehus, NORWAY

## Abstract

**Background and objective:**

The extent to which deep brain stimulation (DBS) can improve quality of life may be perceived as a permanent trade-off between neurological improvements and complications of therapy, comorbidities, and disease progression.

**Patients and methods:**

We retrospectively investigated 123 consecutive and non-preselected patients. Indications for DBS surgery were Parkinson's disease (82), dystonia (18), tremor of different etiology (21), Huntington's disease (1) and Gilles de la Tourette syndrome (1). AEs were defined as any untoward clinical occurrence, sign or patient complaint or unintended disease if related or unrelated to the surgical procedures, implanted devices or ongoing DBS therapy.

**Results:**

Over a mean/median follow-up period of 4.7 years (578 patient-years) 433 AEs were recorded in 106 of 123 patients (86.2%). There was no mortality or persistent morbidity from the surgical procedure. All serious adverse events (SAEs) that occurred within 4 weeks of surgery were reversible. Neurological AEs (193 in 85 patients) and psychiatric AEs (78 in 48 patients) were documented most frequently. AEs in 4 patients (suicide under GPI stimulation, weight gain >20 kg, impairment of gait and speech, cognitive decline >2 years following surgery) were *severe* or worse, at least *possibly* related to DBS and *non* reversible. In PD 23.1% of the STN-stimulated patients experienced *non-reversible (*or *unknown* reversibility) AEs that were at least *possibly* related to DBS in the form of impaired speech or gait, depression, weight gain, cognitive disturbances or urinary incontinence (severity was *mild* or *moderate* in 15 of 18 patients). Age and Hoehn&Yahr stage of STN-simulated PD patients, but not preoperative motor impairment or response to levodopa, showed a weak correlation (r = 0.24 and 0.22, respectively) with the number of AEs.

**Conclusions:**

DBS-related AEs that were *severe* or worse and *non-reversible* were only observed in PD (4 of 82 patients; 4.9%), but not in other diseases. PD patients exhibited a significant risk for *non-severe* AEs most of which also represented preexisting and progressive axial and non-motor symptoms of PD. Mild gait and/or speech disturbances were rather frequent complaints under VIM stimulation. GPI stimulation for dystonia could be applied with negligible DBS-related side effects.

## Introduction

Deep brain stimulation (DBS) has emerged as one of the most effective treatment modalities for movement disorders. There is impartial evidence that alleviation of motor symptoms is associated with a considerable improvement in quality of life in patients with Parkinson's disease (PD) and predominant non-axial motor symptoms or long-term complications from medical treatment [1–3]. Similarly, various types of primary dystonia and tremor show vigorous responses to DBS within the pallidum (commonly referred to as GPI stimulation; GPI, globus pallidus internus) or the ventrolateral thalamus/subthalamic region (commonly referred to as VIM stimulation; VIM, nucleus ventralis intermedius thalami) [4–7].

The surge in quality of life brought on by deep brain stimulation (DBS) is largely determined by cumulated motor improvements balanced against complications of therapy, comorbidities, and disease progression (all referred to as adverse events; AEs). In fact, considering that the overall clinial efficacy of DBS has remained very similar since the advent of well-engineered systems some 20 years ago, the actual gain in quality of life and patient satisfaction is largely determined by the avoidance of AEs.

Whereas AEs related to surgery, such as hemorrhage, infection or surgical revision of hardware are rather obvious and objective, the assessment of neurological AEs (e.g. speech problems) and especially the acquisition of psychiatric AEs (e.g. depression) is more subjective and less consistent. Multiple factors contribute to this. First of all, patients may not voice these kind of complaints or doctors may not pay proper attention to patient complaints. In addition, doctors may not ask patients specifically for possible AEs or may not recognize an unexpected AE. Documentation of AEs may be missing or may only be made when an AE is considered severe enough (threshold effects). Even in clinical trials rating the severity of AEs, for example, semiquantitative rating according to Common Terminology Criteria for Adverse Events (CTCAE), remains somewhat subjective, and standardized rating of patient complaints is almost never performed in the clinical routine. It is often difficult to judge whether or to what degree an AE is directly related to DBS therapy, in particular with regard to preexisting symptoms and comorbidities that tend to worsen over the natural course of the disease. Moreover certain AEs occur with latency under DBS, and short-term assessments in the stimulation “on” and”off” conditions may not suffice to distinguish between DBS-related- and DBS-non related adverse events and such assessments are prone to underestimate the actual rate of DBS-induced AEs. A typical example for this would be axial symptoms in PD. Furthermore, AEs may be itemized using varying and arbitrary categories, i.e. AEs may be assigned to more specific or broader terms. For example, gait disturbances in PD may be subsumed under different terms and more than one of the following items may apply to a given patient: gait problems, postural instability, balance disorder, freezing of gait, festination, start hesition, and falls. This makes it difficult to compare studies and to estimate the overall incidence of DBS-related AEs. Last but not least, the collection and assessment of DBS-related AEs is guided by current knowlegde and is evolving over time. For example, the perception of the behavior of some STN-stimulated PD patients that had been celebrated as gain of initiative, independence, and mobility over a decade ago will nowadays raise red flags with regard to disturbed impulse control.

In fact, the limitations of complete acquisition and proper rating of neurological and psychiatric AEs cannot be overcome by even the most impartial trial methodology. Even blinded and prospective randomized controled trials with tight and independent data monitoring exhibit highly variable rates of neurological and psychiatric AEs, hampering comparisons between studies (cf. Table 1). Low rates of AEs may stem from both a true low incidence or from underreporting. On the other hand, higher rates might be due to 'repeated' reporting, e.g. the documentation of falls and gait disorders for the same patient.

**Table 1:** Reporting of adverse events in prospective multicentric DBS studies for movement disorders.

Author	Pat	SAE	Mortality	Morbidity	Speech	Gait	Depression	Cogniton	Confusion
**Timmermann, 2015**[8]	40	18 / 10	0 (0)	0 (0)	7 (17.5)	14 (35.0)	6 (15.0)	0 (0)	1 (2.5)
**Volkmann, 2014**[9]	62	16 / ND	0 (0)	ND	7 (11.3)	1 (1.6)	4 (6.5)	0 (0)	0 (0)
**Schuepbach, 2013**[10]	124	123 / 68	0 (0)	0 (0)	10 (8.1)	36 (29.0)	33 (26.6)	1 (0.8)	6 (4.8)
**Oderkerken, 2013**[11]	128	ND / ND	0 (0)	1 (0.8)	44 (34.3)	53 (41.4)	10 (7.8)	15 (11.7)	29 (22.7)
**Volkmann, 2012**[6]	40	26 / ND	0 (0)	0 (0)	16 (40.0)	2 (5.0)	2 (5.0)	0 (0)	1 (2.5)
**Okun, 2012**[12]	136	50 / 41	0 (0)	0 (0)	17 (12.5)	49 (36.0)	17 (12.5)	7 (5.1)	22 (16.2)
**Williams, 2010**[3]	183	96 / 65	1 (0.5)	ND	*only SAE specified*				
**Follett, 2010**[13]	299	335 / 160	1 (0.3)	ND	94 (31.4)	407 (136.1)	102 (34.1)	5 (1.7)	70 (23.4)
**Okun, 2009**[14]	52	ND / ND	0 (0)	ND	29 (55.8)	27 (51.9)	40 /76.9)	9 (17.3)	46 (88.5)
**Weaver, 2009**[2]	121	82 / 49	1 (0.8)	ND	19 (15.7)	85 (70.2)	ND	ND	16 (13.2)
**Vidailhet, 2007**[15]	22	3 / 3	0 (0)	0 (0)	*2 stim related AEs*				
**Kupsch, 2006**[16]	40	5 / 5	0 (0)	0 (0)	5 (12.5)	1 (2.5)	ND	ND	1 (2.5)
**Deuschl, 2006**[1]	78	10 / 10	1 (1.3)	0 (0)	8 (10.3)	4 (5.1)	5 (6.4)	3 (3.8)	8 (10.3)
**Vesper, 2002**[17]	129	ND / ND	1 (0.8)	2 (1.6)	3 (2.3)	10 (7.8)	ND	ND	ND
**PD study, 2001**[18]	134	ND / ND	0 (0)	4 (3.0)	2 (1.5)	ND	ND	ND	1 (0.7)
**Schuurman, 2000**[5]	34	ND / ND	1 (2.9)	ND	4 (11.8)	3 (8.8)	ND	ND	ND

For SAE the total number of SAEs (n) and the number of affected patients (pat) is shown. For all other items (speech etc.) the number of affected patients (n) is stated whenever this could be read out of presented data. Otherwise the total number of respective AEs is shown. Only mortality and morbidity due to intracranial hemorrhage was considered. The item 'speech' included dysarthria, hypophonia, and other speech problems, but not impaired word fluency or dysphasia (language problems). The item 'gait' included disturbed balance, freezing of gait, falls, postural instability, festination, start hesitation, and dysequilibrium. The item 'cognition' included memory problems, dysexecution, dysphasia, disturbed word fluency, and mental changes. The item 'confusion' included disorientation, agitation, postoperative psychosis, and delirium. Only patients actually implanted with DBS systems were considered (no intention-to-treat-analyis). ND, not determined or not reported in the study or not ratable from the data presented in the paper.

Monitored trials possess a plethora of data but AEs are usually presented in a rather summarized form and further specifications of AE are sometimes lacking. This lack of detail is mainly for the reason of brevity, although, an independent workup of those substatial data sets together with a more detailed and comprehensive presentation could be addressed in a separate study [19]. We performed complete aquisition and formal rating of all AEs and comorbidities in a non-preselected ('real-world') DBS patient cohort analyzed in a retrospective manner. AEs are broken down with regard to severity, attribution to DBS therapy and reversibility. Data will be presented in a transparent and relatable manner, and, to best of our knowledge, a similar in-depth analysis of AEs has not been undertaken to date.

### Patients and methods

AEs in 123 consecutive and non-preselected patients (56 female; 67 male) who had undergone DBS surgery at our institution between January 1, 2007 and June 30, 2011 were assessed retrospectively including a comprehensive chart review and continuous outpatient documentation. This assured a theoretical follow-up of at least 3 years for all patients until data aquisition. This analysis was performed for the purpose of internal quality control as well as proper patient counseling which should be based on actual AE rates from the treating center and not from the literature. This work is part of a doctoral thesis by one of the authors (K.E.) and was approved by the Medical Faculty of the University of Hamburg. Data entered into the database were analyzed anonymously.

Mean and median age at surgery was 59 and 63 years, respectively (range 17 to 75 years). Twenty patients were aged 70 and older. Mean and median follow-up time was 4.7 years (standard deviation 1.5 years; range 0.7 to 7.3 years). The follow-up period was <12 months for 1 patient (65 year-old patient suffering from ongoing freezing and gait disorder following STN stimulation; follow-up 8 months) and <24 months for 4 patients. In total, this represents a cumulative period of 578 patient-years (4.7 years x 123 patients).

Indications for surgery were Parkinson's disease (82 patients; including 78 patients stimulated bilaterally in the STN, one patient stimulated bilaterally in GPI, and 3 patients stimulated (2 unilaterally) in the VIM); generalized and segmental dystonia (18) with or without associated tremor treated bilaterally in the GPI; essential tremor (14), dystonic tremor (2), symptomatic cerebellar tremor following tumor resection (1) and intention tremor in multiple sclerosis (4) treated with VIM stimulation; Huntington's disease (1; GPI), and Gilles de la Tourette syndrome (1; centre médian-parafascicular nuclei of thalamus, Cm/Pf). For STN-stimulated PD patients disease duration was between 5 and 26 years (mean 13.5; median 14) and mean disease severity was stage 3 according to Hoehn & Yahr. In the preoperative medication "off" state the average UPDRS III (Unified Parkinson's disease rating scale, part III) motor score was 37.1 (median 37; range 14–68) with an average response to levodopa of 44.9% (median 44.4; range 0 (one levodopa non-responsive tremor-dominant PD patient) to 89.8). Average disease duration in dystonia patients treated with GPI stimulation was 14.7 years (median 12; range 3–31) and in tremor patients treated with VIM stimulation it amounted to 18.6 years (median 14; range 2–50). The surgical procedure has been described in a complementing report detailing all surgery-related AEs (Engel et al., submitted).

Adverse events (AEs) were defined as any untoward medical occurrence, clinical sign or patient complaint as well as unintended disease if related or unrelated to the surgical procedures, implanted devices or ongoing DBS therapy. Using a conservative approach AEs included deterioration of preexisting conditions. Abnormal laboratory findings were excluded. AEs were collected from conventional patient records (paper charts) and electronic patient files (Soarian; Siemens, Erlangen, Germany; Dopla system; Carus, Norderstedt, Germany, and 'BIS' system; developed by one of the authors; J.A.K.). A total of 1289 source data documents could be retrieved and were evaluated. Data sources included discharge letters, reports from the outpatient clinics, surgical reports, and other documents such as reports from other institutions or hospitals. Discharge letters following DBS surgery as well as surgical reports were written by one of the authors (W.H.) experienced in deep brain stimulation since 1998. The intention was to keep these documents as complete and consistent as possible prospectively, and all patients were explicitly monitored and interviewed for possible postoperative disturbances, such as impairment of gait or speech, depression, or cognitive deficits.

AEs were grouped into different categories: *neurological*, *psychiatric*, *surgery- and hardware-related*, and *other* AEs. Documentation included the selection of appropriate items for further specification of AEs. For example, neurological AEs included gait disturbance, speech problems etc., and psychiatric AEs included depression, hallucination, confusion etc. (cf. Table 1). We preferred broader terms (e.g. gait disturbance) and added detailed free text descriptions (for example, freezing of gait, postural instability, balance disorder, festination, start hesitation). Similarly psychiatric AEs were summarized in broader items. For example, the mentioning of apathy, diminished initiative or anhedonia in source data was subsumed under depression since there is some overlap or coexistence between these symptoms, although the authors are aware that this results in considerable simplification. Reduced verbal fluency was assigned to cognitive disturbance. This provided a more complete and meaningful assessment and prevented the 'dilution' of complex but related neurological and psychiatric problems by using different entities.

All AEs were rated as to whether these were attributable to DBS or not. If AEs went along with sequelae, such as confusion and deterioration of speech resulting from intracerebral hemorrhage, each of these was documented as a separate AE. For all AEs it was determined whether these represented a serious adverse event (SAEs) according to the criteria set forth by the Food and Drug Administration of the United States of America (http://www.fda.gov). Severity of AEs was graded as *mild*, *moderate*, *severe*, *life-threatening or disabling*, or *death* according to the Common Terminology Criteria for Adverse Events (CTCAE; version 4.0). The relatedness of an AE to surgery or ongoing deep brain stimulation was judged as *unlikely*, *possible*, *probable*, *definite*, or *not related*. In addition, the duration and reversibility of AEs was assessed. Information about preexisting conditions (e.g. speech problems) and comorbidities representing a risk factor (e.g. diabetes for infection) were also recorded.

In DBS-treated PD patients axial symptoms will usually progress due to the natural course of the disease or may become more pronounced (e.g. [20]). During routine follow-up visits, patients were evaluated in the stimulation “on” and “off” conditions as AEs may resolve immediately in the stimulation "off" condition, in particular if these are related to suboptimal electrode placement too close to the internal capsule. This, however, was not observed in any of the patients in the present study. It appears that, for example, impairment of gait or speech that persists in the stimulation "off" condition in PD patients may rather be related to long-term effects of DBS therapy or disease progression. In fact, short-term assessments in the stimulation "on" and "off" states may be misleading and are prone to underestimate the rate of DBS-induced AEs, and proper assessments would require a long wash-out phase to observe potential remission, which had not been performed during routine evaluations. In order to use the most conservative approach, we arbitrarily defined that any worsening of axial symptoms during the postoperative course and within the first 6 months following DBS surgery was rated as *probably* related to DBS, even if there had been statements in source data describing that problems with speech or gait had persisted during short-term trials in the stimulation "off" condition. Worsening of axial symptoms >6 months following surgery was rated as *unlikely* related if not stated otherwise in source data. With regard to an interval of 6 months it was assumed that DBS-related AEs will occur not later than therapeutic effects that are well-known to develop with great latency (e.g. improvement of dystonia). This arbitrary distinction was not made in tremor or dystonia patients. In those patients speech or gait problems were generally rated as *possibly* or *probably* related to DBS therapy if not caused otherwise (e.g. by cervical myelopathy).

In PD axial symptoms and cognitive deficits progress over time. Strictly speaking, any deterioration documented at any time in the post-operative clinical follow-up would have counted as a new AE. However, at routine follow-up visits symptoms are normally not quantified on formal scales and rating had still remained subjective and vague. For this reason, AEs, such as speech problems or cognitive deterioration were only counted once for each patient at the first occurrence within the post-operative follow-up visits. Thus, in some cases, the severity of an AE (e.g. speech problems) may have worsened after the date of first documentation, and eventually the most severe condition was recorded. The reemergence or transient worsening of target symptoms under DBS, for example the recurrence of tremor or the development of tolerance to VIM stimulation, was not rated as an AE. AEs that were *unrelated* to DBS were documented only once. An example for this would be the repeated hospitalization for the treatment of a malignant tumor.

Data were collected by a senior resident (K.E.) experienced in treating movement disorder patients on the ward for several years. However, the author (K.E.) was not involved in the surgical procedures as she was part of a different subspecialty surgical team and thus relatively impartial in documentation. AEs were entered into a relational database developed by one of the authors (J.A.K.) that could be queried with MS Access 2013 (Microsoft Office Access Professional Edition 2013, Microsoft Inc., Seattle, USA). Congruence of the entered AEs with source data (i.e. medical records) was monitored (C.B., J.A.K., W.H.). Since there was no independent external monitoring process the higher CTCAE grade was assigned in case of doubt. Statistical analysis was performed with Sigmastat (Sigmastat 2.03; Systat Software Inc., Chicago, USA).

## Results

### Distribution of AE among patients

**Fig 1:**
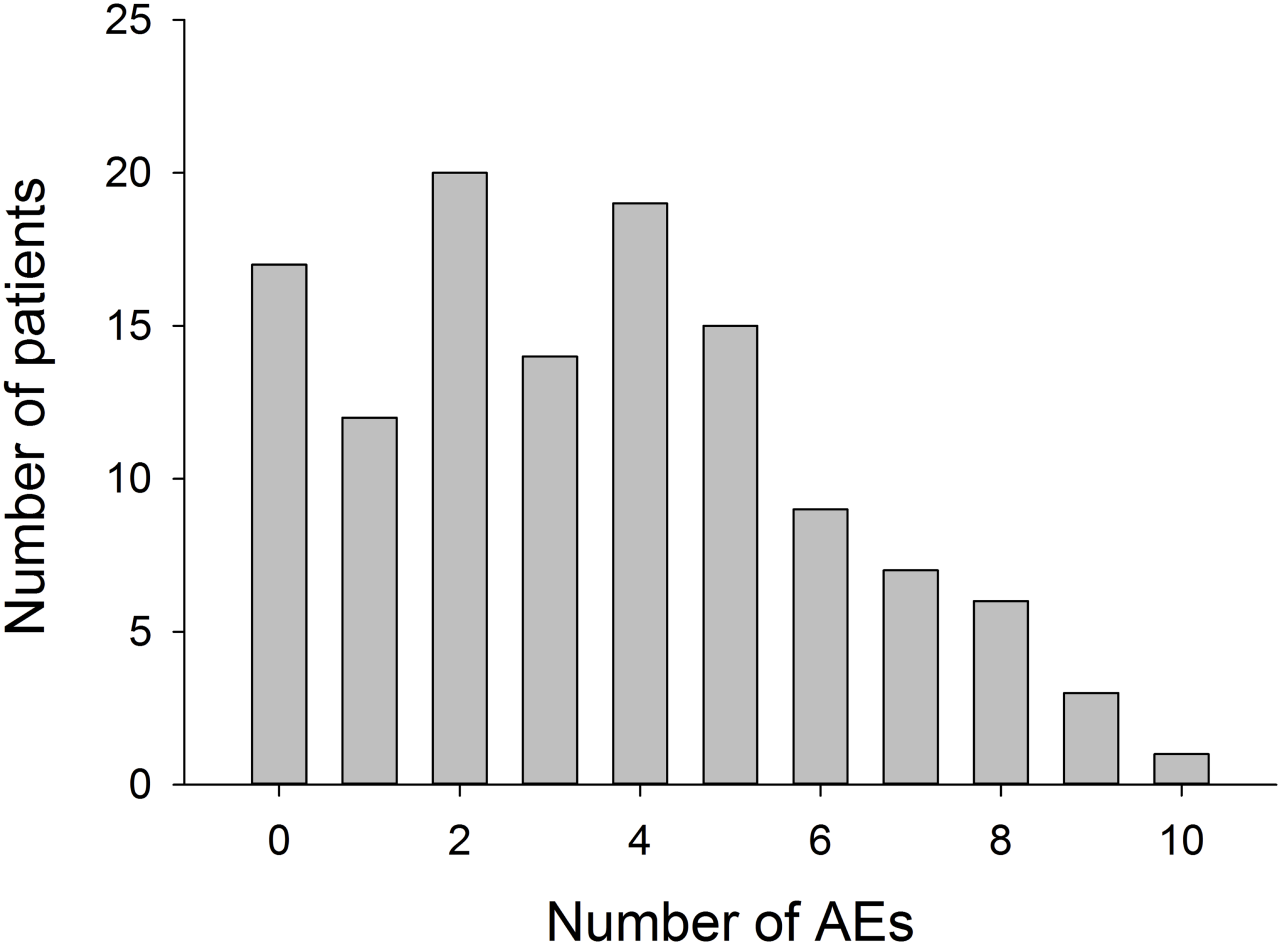
Distribution of AEs (433) among 123 patients. Bars represent the number of patients affected by the same number of AEs. In 17 patients no AEs were noted, in 1 patient 10 AEs occurred.

A total of 433 AEs were retrieved for 106 of 123 patients (86.2%); mean 3.5; median 3; range 0–10 per patient (see Fig 1 for distribution). Neurological and psychiatric AEs were more frequent than AEs related to the surgical procedure or implanted hardware (Table 2). The distribution of the number of AEs per patient is shown in Fig 1. As AEs may represent sequelae from another AE (e.g. neurological deficits from intracerebral hemorrhage) these numbers do not necessarily represent independent AEs. The average number of AEs differed between patients implanted into the STN (3.9 AEs), GPI (3.2), VIM (2.5), and C/P (2). 180 of 433 (41.5%) of the AEs were *mild* or *moderate* and *unlikely* or *not related* to DBS therapy.

**Table 2:** Summary of adverse events.

	AE n (%)	Patients n (%)
**Neurological**	193 (44.6)	85 (69.1)
**Psychiatric**	78 (18.0)	48 (39.0)
**Surgery-related**	23 (5.3)	18 (14.6)
**Other**	139 (32.1)	73 (59.3)
**Total**	433 (100)	106 (86.2)

Summary of all AEs among 123 DBS patients. The number of AEs and the number of patients affected are presented. AEs were grouped into different categories. AEs occurred in 106 of 123 patients. The percentage of affected patients is based on all patients investigated (123). Since patients may have encountered more than one AE or AEs from different categories, the summed-up number of patients (224) exceeds the total number of affected patients (106). Surgery-related AEs included intracranial hemorrhage, infection and wound healing abnormalities as well as AEs involving the implanted hardware.

### Analysis of serious adverse events

**Fig 2:**
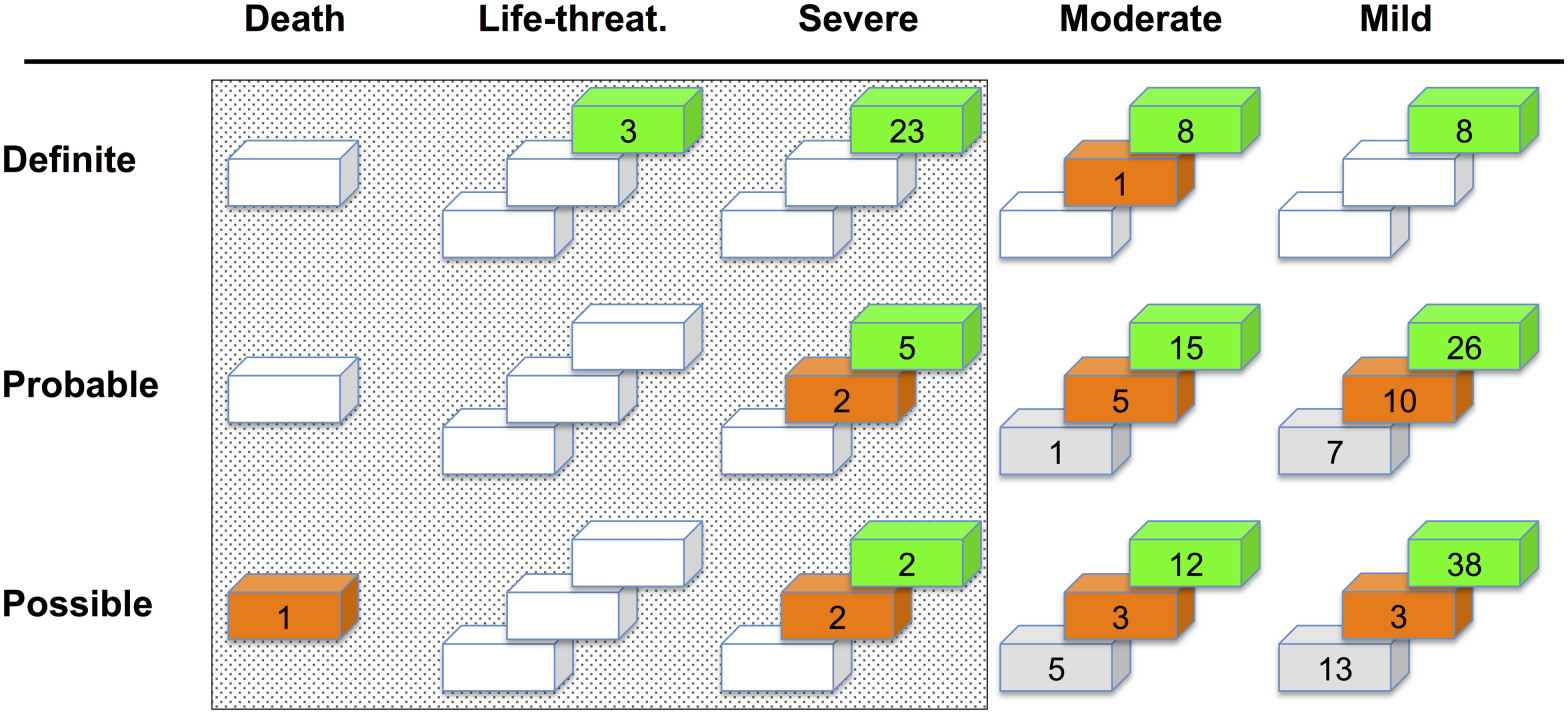
Sum of AEs defined by same severity, reversibility, and attribution to DBS therapy. Green, reversible; orange, non reversible; grey, unknown. The actual number of AEs is presented. The dotted area indicates AEs that were *severe* or worse and at least *possibly* related to DBS therapy and, thus, regarded the most critical. N.B. The number of affected patients may be less than the number indicated because individual patients may have suffered from more than one AE of respective groups (e.g. impairment of gait and speech rated as *mild*, *probably* related and *non-reversible*).

A total of 96 SAEs were documented affecting 59 patients (48%). All SAEs that were at least *possibly* related to DBS and at least of *moderate* severity (n = 38) are specified in Table 3. The majority of SAEs was related to the surgical procedure or implanted devices. All surgery-related SAEs were reversible and will be detailed in a corresponding paper (Engel et al., submitted). In addition, all other SAEs that occurred within the first month of surgery were reversible (Table 3). These included postoperative respiratory complications, akinesia, confusion and one case of postoperative problems with initiation of movements due to a small intracerebral hemorrhage that resolved completely within the weeks following (Fig 2).

**Table 3:** CTC grade of SAE vs relatedness to DBS therapy.

	Definite	Probable	Possible	Unlikely	None
**Death**	–	–	suicide^+^ (18 mo)	–	4
**Life threatening**	akinesia (<1 mo) pneumonia/confusion* (<1 mo) respiratory distress** (<1 mo)	–	–	1	10
**Severe**	akinesia (<1 mo) transient 'paresis'*** (<1 mo) 20 x hardware revision 1 x explantation	3 x injuries (<6 mo) confusion (<1 mo) gait (1 mo) 1 x hardware revision	–	8	28
**Moderate**	2 x intracerebral hemorrhage	gait (3 mo) confusion (3 mo)	gait (16 mo)	2	5

A total of 96 SAE were recorded. These occurred in 59 patients (48% of 123 patients). The actual event is specified for SAE that were at least of 'moderate' severity and at least 'possibly related' to DBS. Numbers in parenthesis indicate the month when the SAE occurred, for example, <1 mo indicates that the AE occurred within the first postoperative month;

*, ICU treatment without intubation;

**, preexisting chronic obstructive lung disease requiring postoperative non-invasive breathing assistance (CPAP) on ICU;

***, 'paresis', initiation of movements was disturbed by ICH, although, with full innervation normal muscle strength could be achieved;

^+^, suicide following GPI stimulation. SAEs that were *unlikely* or *not related* to DBS therapy (58) in most instances (>80%) included 'other' (non-neurological, non-psychiatric, not surgery-related) AEs (cf. Results). In <20% such AEs consisted of neurological or psychiatric AEs occurring >6 months after commencement of DBS leading to admission to a hospital (e.g. infection-associated motor deterioration, incontinence following spine surgery, gait problems, stroke, dysphagia, myelopathy, confusion after 76 months, dysarthria).

The only *non reversible* SAEs that were at least of *moderate* severity and at least *possibly* related to DBS were 2 gait disorders and 1 suicide. The suicide at the age of 52 occurred in a patient suffering from a parkinsonian syndrome diagnosed 11 years prior to surgery. Target symptoms were severe levodopa-induced hyperkinesia as well as tardive dyskinesias that had improved significantly until death 18 months after bilateral GPI electrode implantation. The GPI, instead of the STN, was chosen as the surgical target because of the patient’s history of severe depression.

SAEs that were unlikely related or unrelated to DBS included, for example, two deaths from malignant tumors, one death from subarachnoid hemorrhage, one death from intracerebral hemorrhage after head injury, infections with deterioration of Parkinson's disease several years after surgery, urinary incontinence after spine surgery, decompression for cervical myelopathy and lumbar spinal stenosis, ulnar nerve decompression, treatment for various benign and malignant tumors, pneumonia, pulmonary embolism, and cardivascular events. All of these SAEs occurred >12 months following DBS surgery except for one case of lumbar spine decompression performed after 9 months.

### Incidence of neurological, psychiatric and other AEs

Neurological AEs (related and unrelated to DBS) were observed most frequently and accounted for approximately 45% of all AEs and affected almost 70% of patients (Table 2). Gait disturbances and speech problems (related and unrelated to DBS) were by far the most prevalent AEs (cf. Table 4 for DBS-related AEs and Table 5 (Supplement) for all AEs). Psychiatric AEs (related and unrelated to DBS) represented the second most common AEs (18.0%) and affected approximately 40% of patients (Table 2). Depression and cognitive impairment were observed most frequently (cf. Table 4 for DBS-related AEs and Table 5 (Supplement) for all AEs). *Other* AEs (Table 2) included, for example, postoperative nausea and pain, weight gain, unwanted pregnancy with induced abortion 20 months following GPI stimulation for dystonia, postoperative diarrhea, loosening of teeth following intubation, urinary tract infection, hematuria following catheterization, and pain associated with degenerative spine disease.

**Table 4:** DBS-related neurological and psychiatric adverse events.

Target	STN		VIM		GPI	
Disease	PD		ET		Dystonia	
Patients (n = )	78		14		18	
	n/r	rev	n/r	rev	n/r	rev
**Neurological**						
Gait disturbance	11.5	16.7	35.7	7.1	-	5.6
Speech disturbance	16.7	5.1	14.3	14.3	-	22.2
Akinesia	-	9.0	-	-	-	-
Incontinence	5.1	5.1	-	-	-	-
Dysphagia	-	3.9	-	-	-	-
Hypersalivation	1.3	1.3	-	-	-	-
Eyelid opening apraxia	-	1.3	-	-	-	-
Dysaesthesia	-	-	7.1	14.3	-	-
Paresis	-	1.3	-	-	-	5.6
Neurological other	-	9.0	-	-	-	5.6
						
**Psychiatric**						
Depression	6.4	2.6	-	-	-	-
Cognitive disturbance	3.8	9.0	-	-	-	5.6
Hallucination	-	1.3	-	-	-	5.6
Confusion	-	5.1	-	-	-	11.1
Anxiety	-	-	-	-	11.1	-
Submanic state	-	2.6	-	-	-	-
Psychiatric other	-	6.4	-	-	5.6	-

Numbers indicate incidence of AEs that were at least *possibly* related to DBS (percentage of patients affected); n/r, non reversible AEs or AES of unknown reversibility; rev, reversible AEs

**Table 5:** (Supplement) DBS-related and -unrelated neurological and psychiatric adverse events.

	Total	STN	VIM	GPI	C/P
Patients	123	78	24	20	1
**Neurological**					
Gait disturbance	65 (52.8)	48 (61.5)	14 (58.8)	3 (15.0)	-
Speech disturbance	50 (40.7)	36 (46.2)	8 (33.3)	6 (30.0)	-
Akinesia	12 (9.8)	11 (14.1)	-	1 (5.0)	-
Incontinence	12 (9.8)	11 (14.1)	-	1 (5.0)	-
Dysphagia	9 (7.3)	5 (6.4)	2 (8.3)	2 (10.0)	-
Hypersalivation	8 (6.5)	7 (9.0)	1 (4.2)	-	-
Eyelid opening apraxia	5 (4.1)	5 (6.4)	-	-	-
Dysaesthesia	6 (4.9)	1 (1.3)	4 (16.7)	1 (5.0)	-
Paresis	4 (3.3)	1 (1.3)	1 (4.2)	2 (10.0)	-
Neurological other	22 (17.9)	15 (19.2)	4 (16.7)	3 (15.0)	-
					
**Psychiatric**					
Suicide	1 (0.8)	-	-	1 (5.0)	-
Depression	19 (15.4)	12 (15.4)	4 (16.7)	2 (10.0)	1
Cognitive disturbance	20 (16.3)	17 (21.8)	1 (4.2)	2 (10.0)	-
Hallucination	10 (8.1)	8 (10.3)	-	2 (10.0)	-
Confusion	8 (6.5)	5 (6.4)	1 (4.2)	2 (10.0)	-
Impuls control disorder	3 (2.4)	3 (3.8)	-	-	-
Anxiety	2 (1.6)	-	-	2 (10.0)	-
Submanic state	2 (1.6)	2 (2.6)	-	-	-
Psychiatric other	13 (10.6)	7 (9.0)	1 (4.2)	5 (25.0)	-

Numbers indicate incidence of DBS-related and -unrelated AEs (in parenthesis percentage of patients affected); C/M, centre médian-parafascicular nuclei of thalamus. Other neurological AEs (Table 4) included AEs such as stroke (after 79 months), facial palsy (after 6 months), ulnar palsy, disturbed fine motor skills (e.g. writing), diplopia (the latter 3 occurring after >32 months), other visual problems (e.g. macular dystrophy), symptoms resembling restless legs syndrome, postural abnormalities (e.g. Pisa syndrome), and others. Other psychiatric AEs included sleep disturbances and nightmares, fatigue, somatoform disorder, convulsive sobbing, personality disorder trait, and tension.

### Severity and reversibility of AE vs attribution to DBS surgery and ongoing DBS therapy

In Fig 2, the severity of AEs is plotted against their attribution to DBS therapy. Only AEs that were at least *possibly* related to DBS therapy are considered. In the third dimension, the reversibility of AEs is shown.

Those AEs that were both rated as *severe* or worse and at least *possibly* related to DBS were regarded as the most critical (dotted area in Fig 2). The majority of such 'critical' AEs consisted of surgery- and hardware-related complications and all of these were reversible (cf. 'Analysis of serious adverse events'). Such 'critical' AEs were *not reversible* in only 4 patients: one patient committed suicide (described above), one female PD patient experienced weight gain of >20 kg, in one patient, deterioration of gait and speech impaired activities of daily life within six months of DBS surgery, and in one patient progressive cognitive disturbances documented >2 years following surgery were rated as possibly related to DBS because (initially reversible) confusion had already occurred in the postoperative period.

Most *mild* or *moderate* AEs that were at least *possibly* related to DBS therapy were neurological and psychiatric AEs also representing well-known and often preexisting axial problems and other comorbidities in PD patients, and these will be detailed in the following.

Speech problems were *mild* (66.7%) or *moderate* (25.0%) in most instances with 1 case of *severe* dysarthria in STN-stimulated PD patients. Impairment of speech that had occurred within the first 6 months (44.4% of speech problems) was *mild* in the vast majority of cases (81.3%). In STN-stimulated PD patients the actual risk for *non-reversible* (or reversibility *unknown)* impairment of speech that was at least *possibly* related to DBS was 16.7% (Table 4). With regard to speech problems that had been noticed in STN-stimulated PD patients later than 6 months following STN surgery, it was *reversible* in only one case. Two dystonic tremor patients complained about *mild* speech and gait problems within 6 months from surgery requiring adjustment of stimulator settings. Four ET patients experienced speech problems in conjunction with the requirement to increase stimulator settings >6 months after surgery. Only 1 dystonia patient reported *mild* speech problems, and these were *reversible* within 6 months from surgery. Speech problems in all the other dystonia patients were documented >29 months after surgery and were *mild* and mostly involved the mentioning of short episodes of slurred speech in the evening when being exhausted.

In STN-stimulated PD patients the actual risk for *non reversible* (or reversibility *unknown)* impairment of gait that was at least *possibly* related to DBS was 11.5% (Table 4). Two dystonic tremor patients and 2 ET patients recognized some difficulties with walking within 6 months from surgery and before stimulator settings had been optimized. After >6 months 4 other ET patients mentioned at least *possibly* related gait problems that were associated with the need to increase stimulation amplitudes in order to maintain sufficient tremor suppression. Unsteady gait (*possibly* related) occured in only 1 dystonia patient after 16 months and resolved with normalization of blood pressure after ramipril was discontinued. *Severe* gait disturbances that were *unlikely* related to DBS developed in 3 patients: one patient with progression of MS after >24 months, one ET patient after >4 years due to cervical myelopathy, and one PD patient after spine surgery.

Postoperative confusion and hallucinations were *reversible* in all cases. Two patients developed a postoperative submanic state (mild and moderate) that resolved after adaptation of medication and stimulation (one of both cases was reported in [21]).

Depression was *mild* (57.9%) or *moderate* (31.6%) in most patients, except for *severe* and *life-threatening* depression in 2 patients one of whom later committed suicide (Tables 3 and 4). Both patients (one PD patient and one patient with hyperkinetic disorder of unknown etiology) were stimulated in the GPI. In STN-stimulated PD patients the actual risk of *non reversible* (or reversibility *unknown)* depression that is at least *possibly* related to DBS was 6.4% (Table 4).

Cognitive decline was *mild* or *moderate* in all but one patient (dementia >24 months following STN surgery). Transient cognitive impairment occurred in conjunction with both intracerebral hematomas. In 40% of the cases cognitive declines were observed >24 months after surgery. In 5 cases cognitive disturbances were documented within the first 6 months following STN surgery, of which 3 were completely *reversible*. In STN-stimulated PD patients the actual risk for *non reversible* (or reversibility *unknown)* cognitive decline that is at least *possibly* related to DBS was 3.8% (Table 4).

In addition, urinary incontinence (4 patients) and weight gain (1 patient) were found among AEs that were *non reversible* or of *unknown* reversibility affecting 5.1% and 1.3% of STN-stimulated PD patients, respectively.

Taking into account that patients may be affected by more than one AE, 18 of 78 (23.1%) STN-stimulated PD patients experienced *non-reversible (*or *unknown* reversibility) AEs that were at least *possibly* related to DBS in the form of impaired speech or gait, depression, weight gain, cognitive disturbances or urinary incontinence.

In ET, 5 of 14 patients were affected by speech and/or gait disturbances. In addition, both patients receiving VIM stimulation for dystonic tremor experienced dysarthria and gait disturbances that were at least *possibly* related to DBS. In 5 patients receiving unilateral VIM stimulation (1 ET, 2 PD, 2 MS) only one AE (gait disturbance in MS) was rated as *non-reversible* and *possibly* related to DBS.

### Analysis of possible risk factors

Whereas mean and median age of patients with 0 to 5 AEs (n = 97) was 57.9 and 61 years, respectively, this was higher in patients with 6 to 10 AEs (63.3 and 66 years, respectively; n = 26). This difference came close to but missed statistical significance (p = 0.05; Mann Whitney rank sum test). Age was weakly correlated with the number of AEs (r = 0.24; r^2^ = 0.058; p = 0.007). Both genders were similarly affected by AEs (male 228 of 433 AEs; 52.6%).

In STN-stimulated PD patients disease duration or disease severity according to the preoperative UPDRS III motor score in the medication "off" state did not reveal a positive correlation with the number of AEs that had been rated at least *possibly* related to DBS (Spearman's rank coefficient, r< 0.2; p> 0.5). A weak correlation was found between disease severity according to Hoehn&Yahr stages and the number of AEs (Spearman's rank coefficient r = 0.22; p< 0.052). In dystonia patients treated with GPI stimulation and in tremor patients treated with VIM stimulation there was no correlation between disease duration and the number of AEs (r< 0.1; p> 0.05).

## Discussion

This study provides a comprehensive retrospective long-term analysis of AEs representing complications of DBS surgery and ongoing therapy as well as untoward events related to comorbidities and progression of the underlying diseases. AEs are unraveled in multiple dimensions, i.e. with respect to severity, relatedness to DBS therapy, and reversibility, and all critical AEs are detailed in a relatable manner. To the best of our knowledge, a similarly detailed assessment has not been published for DBS patients thus far.

There was no mortality or persistent morbidity from the surgical procedure, and all surgery-related AEs were *reversible* and resolved without sequelae. One suicide occurred under GPI stimulation after 18 months. In this patient the GPI instead of the STN nucleus was chosen because of the patient’s past history of severe depression. Although suggested previously, more recently there has been doubt whether DBS increases the risk of suicide [22, 23]. Only 3 other *non-reversible* AEs were rated as *severe* or worse and at least *possibly* related to DBS involving gait and speech disorder, cognitive decline >2 years following surgery and weight gain in one patient each.

The majority of AEs was documented during ongoing DBS therapy covering a period of 578 patient-years (4.7 years mean/median follow-up). The list of AEs is headed by speech problems and gait disorders, and the most common psychiatric AEs were depression and cognitive decline. These AEs also represent cardinal symptoms and comorbidities of the underlying diseases (e.g. gait and speech problems and depression in Parkinson's disease; gait and speech problems in essential tremor) [19, 20, 24–29]. For this reason alone it is difficult to assess their relatedness to DBS therapy. In Parkinson's disease, we arbitrarily chose to attribute worsening of axial symptoms as *probably* related to DBS therapy when this occurred within 6 months. A period of 6 months provides a margin of safety as even long-latency therapeutic effects of DBS (for example, improvement of dystonia) usually evolve much earlier. In clinical studies with follow-up periods of >6 months the presumed relatedness of AEs to DBS therapy should be reported, especially when there is no control group.

Voice and speech disturbances are preexisting in most PD patients prior to STN surgery. These deficits may deteriorate with the natural course of the disease and may worsen under STN stimulation [19, 20, 25, 27]. Our data suggest that the risk of *non reversible mild* or *moderate* impairment of speech within six months of STN stimulation is approximately 17%. Rates in monitored trials are very variable and range between <10% and >50% (Table 1). The exact phenotypic characteristics associated with impaired speech intelligibility and the actual functional impairments caused by DBS therapy still require further elucidation and exhibit high individual variability [30–39]. Whereas reduced volume is observed in almost all PD patients slurred speech has been regarded rather as a side effect of DBS therapy [38].

Although gait disturbances are usually pre-existent and progressive in PD, deterioration of gait (e.g. difficulties walking or freezing of gait) within the first days or weeks following DBS surgery may resolve with time (e.g. due to resolution of a microlesioning effect) and after stimulator settings and medication have been adjusted. In the long run preoperative gait disturbances may improve in STN-stimulated PD patients, in particular if these had proven to be levodopa-responsive [29, 40–46]. Nonetheless, gait problems may persist in a proportion of patients. Our data suggest that about 12% of STN-stimulated PD patients exhibit gait disturbances within the first 6 months of STN stimulation that were *non reversible* (or reversibility *unknown)*. But even with an uneventful postoperative course and despite improvement of gait over a period of several years this does not prevent most PD patients from developing gait problems and falls later on due to disease progression [19, 20, 25, 27–29, 47–50]. A meta-regression performed by St. George et al. revealed that despite initial improvements in balance and gait compared to the preoperative state, the long-term application of STN stimulation (less with GPI stimulation) resulted in a progressive decline of balance and gait in PD patients [29]. In monitored trials the frequency of gait disturbances in PD patients ranges between 5% and >100% (Table 1). This illustrates the difficulties in gathering, rating and evaluating gait problems, in particular if these coincide with preexistent and progressive PD symptoms (Table 1).

In contrast to PD patients, in tremor and dystonia patients speech and gait problems were always *mild*. There were no other *non reversible* AEs that could at least *possibly* be related to DBS therapy indicating that GPI stimulation for dystonia is very well tolerated and could be applied virtually without side effects.

Bilateral stimulation in the ventrolateral thalamus and subthalamic area is associated with an increased risk of gait and speech disturbances (e.g. [51]). These only occurred in bilaterally stimulated patients in the present series, but not in patients receiving unilateral VIM stimulation (with the exception of 1 MS patient with preexisting gait disorder). The underlying mechanisms for the development of speech and gait disturbances under VIM stimulation have not been resolved yet. The development of tolerance (or habituation) associated with the need to increase stimulation amplitudes for the long-term suppression of tremor in some patients as well as the progression of pre-existing gait and speech abnormalities in ET and dystonic tremor patients appear to play a role [52–65].

In several studies it was found that average depression scores among STN-stimulated PD patients were improved compared to the preoperative state [66–70]. However, preoperative depression may temporarily be aggravated by the reduction of dopaminergic medication in the postoperative phase and depression may improve again after long-term adjustments of stimulation and medication have been made. This explains the fact that the reported rates of depression in monitored clinical trials covering the postoperative phase may be relatively high (up to 77%; Table 1). In the present study, for STN-stimulated PD patients the risk of *non reversible* (or reversibility *unknown)* depression that was at least *possibly* related to DBS was 6.4%.

Immediate cognitive deficits after DBS procedures may be observed and the risk appears to be increased in PD patients already exhibiting cognitive impairments at baseline [71, 72]. Usually postoperative decline is worst in the first months following STN surgery and may improve in the ensuing months [73, 74]. Our data indicate that even with unsuspicious cognitive testings prior to STN surgery about 4% of patients may be affected by *non reversible* cognitive decline that was rated at least *possibly* related to DBS. It is unclear to what extent surgery or anesthesia as opposed to high-frequency stimulation of the STN contribute to cognitive decline [14, 73]. On the other hand, also improvements of cognitive aspects under STN stimulation have been observed [69, 75–77]. Cognitive impairments that have rather consistently been attributed to STN surgery and STN stimulation are disturbances of verbal fluency, memory and executive functioning [14, 28, 67, 69, 70, 73, 78–84], and also in our patients affected by cognitive deficits these represented the most common items.

Rather surprising was the frequency of postoperative (worsening of preexisting) urinary incontinence under STN stimulation occurring within the first 6 months following surgery. There may be underreporting of this AE in previous reports. Other patients, however, may also experience improved bladder control [85–87]. All our patients exhibiting postoperative urinary incontinence (2 female, 2 male) had perioperatively received transurethral indwelling catheters involving uncomplicated catheterization. In all patients complaints or signs of urinary incontinence were already present prior to initiation of high-frequency stimulation of the STN. This is suggestive of microlesioning effects or residual effects of anesthesia.

Overall approximately 25% of the PD patients experienced *non-reversible (*or *unknown* reversibility) AEs that were at least *possibly* related to STN stimulation in the form of impaired speech or gait, depression, weight gain, cognitive disturbances or urinary incontinence. This number appears relatively high but seems to be in accordance with the clinical experience that approximately 1 in 4 STN-stimulated PD patients requires increased attention to one or several of these problems. However, one has to take into consideration that in the majority of cases those AEs were *mild*, most conditions were preexisting (e.g. impaired speech or gait, depression), and the overall quality of life in these patients may still be improved by DBS, and most patients would choose to undergo STN surgery again (different questionnaires about quality of life and satisfaction with therapy; CKEM, AG et al., unpublished data).

### Strengths and limitations of the study

We investigated a non preselected ('real world') patient cohort involving the most common diseases treated by DBS in the most common surgical targets. Thus, this study is not charged with the unavoidable selection bias of prospective studies recruiting patients according to defined inclusion criteria. Our cohort is likely to represent patient populations similar to those of many DBS centers. All AEs were formally rated and presented in a transparant and relatable manner that, to the best of our knowledge, has not been performed for DBS patients to date.

The evaluation of AEs occurring under ongoing DBS therapy was facilitated by the fact that all AEs related to surgery were reversible. Only 2 patients showed transient neurological deterioration due to small intracerebral hemorrhages. In addition, none of the implanted electrodes had to be revised because of misplacement, lack of efficacy or intolerable side effects. Thus, almost all neurologcial and psychiatric AEs that were rated at least *possibly* related to DBS therapy can be attributed to ongoing stimulation performed in a standard manner as opposed to directly caused by the surgery itself (except for microlesioning effects that cannot be ruled out). In other words, a higher incidence of surgery- or lead-related complications with neurological and psychiatric sequelae would have increased the actual rate of AEs and made it more difficult to determine to what extent these are related to DBS therapy or complications of surgery itself (e.g. suboptimal lead placement). AE rates in the present study are rather representative for uncomplicated postoperative courses.

Limitations of our study are its retrospective design and lack of a non-treated control group. Although the frequency of neurological and psychiatric AEs in the present series was higher than in several other—even monitored—studies (Table 1), an underreporting of, for example, sleep disturbances, pain, and obstipation in PD patients may be present in our series. The numerous factors responsible for variable reporting of AEs have been addressed in detail in the introduction, and it would be presumptuous to assume the present study was completely unaffected by those factors.

In the present study AEs were first collected in broader terms (e.g., speech or gait) and specified later in order to prevent diluted rates and to generate meaningful numbers that can be used for comparison, patient counseling and informed consent. Patient follow-up was comparably long (cf. Table 1). The longer the follow-up period the higher is the proportion of AEs that are not related to DBS therapy (e.g. disease progression and comorbidities) and the more important it is to properly assess the relatedness to DBS.

### Conclusion

Taken together, the present study provides a detailed and relatable analysis of AEs occurring in patients undergoing DBS surgery and long-term therapy. The assessment of neurological and psychiatric AEs, representing the most frequently recorded AEs, is limited by patient- and physician-related factors and also by the fact that there are no standardized procedures for the collection, evaluation and presentation of such data. This results in highly variable rates in the literature. AEs should be collected in rather broad terms and rated with regard to severity, reversibility and relatedness to DBS therapy as performed in the present study. It should be mandatory for clinical DBS studies to present actual details about critical AEs comprising those that are rated as *severe* or worse and at least *possbily related* to DBS in a comprehensive and relatable manner. In particular for axial symptoms in STN-stimulated PD patients the rating of *relatedness* and potential *reversibility* of AEs is equivocal. This is mainly due to gaps in knowledge (1) about the kinetics with which different AEs develop under DBS and (2) about the kinetics of the progression of different symptoms of the underlying (neurodegenerative) disease in a given patient. All serious adverse events (SAEs) that occurred within 4 weeks of surgery were reversible. DBS-related AEs that were *severe* or worse and *non-reversible* were only observed in PD affecting 4 of 82 patients (4.9%). PD patients exhibited a significant risk for *non-severe* AEs. Most of these were axial and non-motor symptoms that slightly preexist in all PD patients and also represent the most relevant long-term problems. Age and Hoehn&Yahr stage of STN-simulated PD patients, but not preoperative motor impairment or response to levodopa, showed a weak correlation (r = 0.24 and 0.22, respectively) with the number of AEs. Mild gait and/or speech disturbances were rather frequent complaints under VIM stimulation. GPI stimulation for dystonia could be applied with negligible DBS-related side effects.
